# Supplementary material for: Gepants for Acute and Preventive Migraine Treatment: A Narrative Review
Source: Brain Sci. 2022 Nov 24;12(12):1612. doi: 10.3390/brainsci12121612 (PMC9775271; doi:10.3390/brainsci12121612)
Supplement: Supplementary file 1 [file brainsci-12-01612-s001.zip › brainsci-2017558-supplementary.pdf]

Gepants For Acute And Preventive Migraine Treatment: A Narrative Review

SUPPLEMENTARY MATERIAL

Supplementary Material 1 – Methodology

Supplementary Material 2 – Computed pharmacological properties to the second and third generation of gepants

Supplementary Material 3 – Summary of clinical trials related to rimegepant, ubrogepant, atogepant, and zavegepant

Supplementary Material 4 – Network meta-analyses comparing the efficacy of lasmiditan, rimegepant, and ubrogepant published in the literature

Supplementary material 1 – Methodology

METHODS

Search Strategy

We searched six databases to locate the studies on migraine and small molecule calcitonin gene-related peptide receptor antagonists published from 2012 to June 2022 in electronic form. Excerpta Medica (Embase), Google Scholar, Latin American & Caribbean Health Sciences Literature (Lilacs), Medline, Scientific Electronic Library Online (Scielo), and Science Direct were searched. Search terms were “rimegepant, ubrogepant, atogepant, zavegepant, gepant” These terms were combined with “migraine”. Publications in English and Spanish were included in the search. Additional data was included from abstracts presented at the American Academy of Neurology, International Headache Society, and American Headache Society meetings.

(migraine) AND (rimegepant)	("migrain"[All Fields] OR "migraine disorders"[MeSH Terms] OR ("migraine"[All Fields] AND "disorders"[All Fields]) OR "migraine disorders"[All Fields] OR "migraine"[All Fields] OR "migraines"[All Fields] OR "migraine s"[All Fields] OR "migraineous"[All Fields] OR "migrainers"[All Fields] OR "migrainous"[All Fields]) AND ("rimegepant sulfate"[Supplementary Concept] OR "rimegepant sulfate"[All Fields] OR "rimegepant"[All Fields])
(migraine) AND (ubrogepant)	("migrain"[All Fields] OR "migraine disorders"[MeSH Terms] OR ("migraine"[All Fields] AND "disorders"[All Fields]) OR "migraine disorders"[All Fields] OR "migraine"[All Fields] OR "migraines"[All Fields] OR "migraine s"[All Fields] OR "migraineous"[All Fields] OR "migrainers"[All Fields] OR "migrainous"[All Fields]) AND ("ubrogepant"[Supplementary Concept] OR "ubrogepant"[All Fields])
(migraine) AND (atogepant)	("migrain"[All Fields] OR "migraine disorders"[MeSH Terms] OR ("migraine"[All Fields] AND "disorders"[All Fields]) OR "migraine disorders"[All Fields] OR "migraine"[All Fields] OR "migraines"[All Fields] OR "migraine s"[All Fields] OR "migraineous"[All Fields] OR "migrainers"[All Fields] OR "migrainous"[All Fields]) AND ("atogepant"[Supplementary Concept] OR "atogepant"[All Fields])
(migraine) AND (zavegepant)	("migrain"[All Fields] OR "migraine disorders"[MeSH Terms] OR ("migraine"[All Fields] AND "disorders"[All Fields]) OR "migraine disorders"[All Fields] OR "migraine"[All Fields] OR "migraines"[All Fields] OR "migraine s"[All Fields] OR "migraineous"[All Fields] OR "migrainers"[All Fields] OR "migrainous"[All Fields]) AND "zavegepant"[All Fields]
(migraine) AND (gepant)	("migrain"[All Fields] OR "migraine disorders"[MeSH Terms] OR ("migraine"[All Fields] AND "disorders"[All Fields]) OR "migraine disorders"[All Fields] OR "migraine"[All Fields] OR "migraines"[All Fields] OR "migraine s"[All Fields] OR "migraineous"[All Fields] OR "migrainers"[All Fields] OR "migrainous"[All Fields]) AND ("calcitonin gene related peptide receptor antagonists"[Pharmacological Action] OR "calcitonin gene related peptide receptor antagonists"[MeSH Terms] OR ("calcitonin"[All Fields] AND "gene related"[All Fields] AND "peptide"[All Fields] AND "receptor"[All Fields] AND "antagonists"[All Fields]) OR "calcitonin gene related peptide receptor antagonists"[All Fields] OR "gepant"[All Fields])

**Supplementary material 2 – Computed pharmacological properties to the second and third generation of gepants**				
Drug	Rimegepant	Ubrogepant	Atogepant	Zavegepant
Physicochemical Properties				
Formula	$C_{28}H_{28}F_2N_6O_3$	$C_{29}H_{26}F_3N_5O_3$	$C_{29}H_{23}F_6N_5O_3$	$C_{36}H_{46}N_8O_3$
Molecular weight (150–500 g/mol)	534.56	549.54	603.52	638.80
Fraction Csp3 (0.25–1.0)	0.36	0.34	0.34	0.50
Num. rotatable bonds (0-9)	5	6	6	9
Num. H-bond acceptors (≤10)	8	8	11	6
Num. H-bond donors (≤5)	2	2	2	3
Topological polar surface area (20-130 Å²)	119.13	104.29	104.29	120.67
Lipophilicity				
Consensus Log Po/w (0.7–5.0)	3.18	3.29	4.20	3.11
Water Solubility				
Log S (ESOL) (- 6–0)	-4.67	-5.14	-5.65	-5.48
Class	Moderately soluble	Moderately soluble	Moderately soluble	Moderately soluble
Pharmacokinetics				
Gastrointestinal absorption	High	High	High	High
Blood-brain-barrier permeant	No	No	No	No
Druglikeness				
Bioavailability score	0.55	0.55	0.55	0.17
Medicinal Chemistry				
Synthetic accessibility [1(very easy) – 10 (very difficult)]	5.20	5.27	5.40	5.49
Other pharmacokinetic characteristics extracted outside SwissADME (references below)				
Human CGRP receptor binding affinity (pM)	32.9	70	15-26	23
Tmax	1.5h	1.5h	1-2h	15-20 minutes
Bioavailability	64%	40-75% (under study)	under study	<30% (under study)
Volume distribution	120L	350	292	Under study
Plasma-protein binding	96%	87%	98.2%	Under study
Metabolism	Hepatic (CYP3A4)	Hepatic (CYP3A4)	Hepatic (CYP3A4)	Under study
Elimination half-life	11h	5-7h	11h	Under study
Excretion	Feces (78%) > urine (24%)	Feces (42%) > urine (6%)	Feces (81%) > urine (8%)	Under study

The normal range provided is desirable for oral drugs. Consensus Log Po/w is the average of iLOGP, XLOGP3, WLOGP, MLOGP, and SILICOS-IT.

References:

1) https://www.accessdata.fda.gov/drugsatfda_docs/nda/2020/212728Orig1s000PharmR.pdf
2) Moore E, Fraley ME, Bell IM, Burgey CS, White RB, Li CC, Regan CP, Danziger A, Stranieri Michener M, Hostetler E, Banerjee P, Salvatore C. Characterization of Ubrogepant: A Potent and Selective Antagonist of the Human Calcitonin Gene–Related Peptide Receptor. J Pharmacol Exp Ther 2020:jpet.119.261065.
3) Moreno-Ajona D, Pérez-Rodríguez A, Goadsby PJ. Small-molecule CGRP receptor antagonists: A new approach to the acute and preventive treatment of migraine. Medicine in Drug Discovery. 2020 Sep 1;7:100053.
4) Mercer SE, Chaturvedula PV, Conway CM, Cook DA, Davis CD, Pin SS, Macci R, Schartman R, Signor LJ, Widmann KA, Whiterock VJ, Chen P, Xu C, Herbst JJ, Kostich WA, Thalody G, Macor JE, Dubowchik GM. Azepino-indazoles as calcitonin gene-related peptide (CGRP) receptor antagonists. Bioorg Med Chem Lett. 2021 Jan 1;31:127624. doi: 10.1016/j.bmcl.2020.127624. Epub 2020 Oct 21. PMID: 33096162.
5) Daina A, Michielin O, Zoete V. SwissADME: a free web tool to evaluate pharmacokinetics, drug-likeness and medicinal chemistry friendliness of small molecules. Sci Rep. 2017 Mar 3;7:42717. doi: 10.1038/srep42717. PMID: 28256516; PMCID: PMC5335600.

Supplementary Material 3 – Summary of clinical trials related to rimegepant, ubrogepant, atogepant, and zavegepant					
ClinicalTrials.gov Identifier	Title	Condition	Estimated/ actual enrollment (participants)	Estimated/ actual study completion date	Brief summary
RIMEGEPANT					
NCT01430442	Dose-ranging study of rimegepant (bms-927711) for the acute treatment of migraine	Migraine; acute treatment of migraine	1026	May 2012	The primary purpose of this study is to evaluate the efficacy of rimegepant (BMS-927711) compared with placebo in the acute treatment of migraine as measured by pain freedom at 2 hours post-dose using a four-point numeric rating scale while identifying an optimal dose to support the Phase 3 clinical trials.
NCT03235479	Safety and efficacy study in adult subjects with acute migraines	Migraine, with or without aura	1485	January 2018	The purpose of this study is to compare the efficacy of BHV-3000 (rimegepant) versus placebo in subjects with acute migraines
NCT03237845	Safety and efficacy in adult subjects with acute migraines	Migraine, with or without aura	1499	January 2018	The purpose of this study is to compare the efficacy of BHV-3000 (rimegepant) versus placebo in subjects with Acute Migraines
NCT03461757	Trial in adult subjects with acute migraines	Migraine, with or without aura	1811	October 2018	The purpose of this study is to compare the efficacy of BHV-3000 (rimegepant ODT) versus placebo in subjects with acute migraines.
NCT03732638	Efficacy and safety trial of rimegepant for migraine prevention in adults	Migraine	1591	February 2021	The purpose of this study is to compare the efficacy of BHV-3000 (rimegepant) to placebo as a preventive treatment for migraine, as measured by the reduction in the number of migraine days per month.
NCT04574362	Safety and efficacy trial of bhv3000 (rimegepant) 75 mg for the acute treatment of migraine	Acute migraine	1648	December 2021	This trial is to determine whether BHV3000 (rimegepant) 75mg is safe and effective as a treatment for acute migraine in Chinese and Korean patients
NCT04629950	Rimegepant in moderate plaque-type psoriasis	Psoriasis	30	December 2022	The purpose of this study is to examine the use of rimegepant for the treatment of moderate plaque-type psoriasis.
NCT05127486	A study of galcanezumab (ly2951742) in adult participants with episodic migraine (challenge-mig)	Migraine; episodic migraine	700	December 2022	The purpose of this study is to assess whether galcanezumab is superior to rimegepant in the prevention of migraine in participants with episodic migraine. The study duration will be approximately 6 months.
NCT04860713	An efficacy and safety of proprietary formulations of oral ketamine + aspirin	Pain	90	December 2022	To compare the analgesic efficacy and rates of side effects of a proprietary formulation of orally administered aspirin and ketamine to rimegepant for pain management in adult

	in treatment of acute headache				emergency department patients presenting to the emergency department with acute headache.
NCT04649242	Randomized study in children and adolescents with migraine: acute treatment	Pediatric migraine	1440	January 2023	The purpose of this study is to test the safety and efficacy of BHV-3000 versus placebo in the acute treatment of moderate or severe migraine in children and adolescents.
NCT05248997	Safety and efficacy of bhv-3000 (rimegepant) orally disintegrating tablet for the acute treatment of chronic rhinosinusitis	Chronic rhinosinusitis with and without nasal polyps	200	February 2023	The purpose of this study is to compare the efficacy and safety of rimegepant versus placebo in the acute treatment of chronic rhinosinusitis with and without nasal polyps.
NCT05262517	Safety and efficacy of bhv-3000 (rimegepant) orally disintegrating tablet for acute treatment of temporomandibular disorders	Temporomandibular disorders	200	March 2023	The purpose of this study is to compare the efficacy and safety of rimegepant versus placebo in the acute treatment of temporomandibular disorders, which are medical conditions involving the temporomandibular joint (the joint connecting the jawbone to the skull) and surrounding muscles and tissues.
NCT03941834	Trial for treatment refractory trigeminal neuralgia	Trigeminal neuralgia	60	May 2023	The purpose of this study is to evaluate the efficacy of BHV3000 compared to placebo for subjects with treatment-refractory trigeminal neuralgia between the two-week treatment phases.
NCT05264714	Cluster headache treatment with rimegepant	Cluster headache	10	June 2023	The purpose of this research is to explore the efficacy of rimegepant as a preventative therapy for cluster headaches.
NCT05207865	Safety and tolerability study of daily dosing rimegepant in episodic migraine prevention	Migraine; episodic migraine	125	September 2023	The purpose of this study is to further evaluate the long-term safety and tolerability of daily dosing of rimegepant for the prevention of episodic migraine.
NCT05217927	Efficacy and safety study of rimegepant in episodic migraine prevention with multiple dosing regimens	Migraine	660	September 2023	The purpose of this study is to compare the efficacy and safety of daily and every other day dosing of rimegepant to placebo as a preventive treatment for episodic migraine.
NCT04743141	Long-term safety study of rimegepant in pediatric subjects for the acute	Acute treatment of migraine	600	October 2023	The purpose of this study is to test the long-term safety of rimegepant in the acute treatment of moderate or severe migraine in children and adolescents (≥ 6 to < 18 years of age).

	treatment of migraine				
NCT05399459	Efficacy and safety study of rimegepant for the acute treatment of migraine in japanese subjects	Migraine	795	January 2024	This study is being conducted to determine the appropriate dose of rimegepant in Japanese subjects, as well as to evaluate the efficacy, safety, and tolerability of rimegepant in Japanese subjects for the acute treatment of migraine.
NCT05399485	Efficacy and safety study of rimegepant for migraine prevention in japanese subjects	Migraine	490	October 2024	This study is being conducted to evaluate the efficacy, safety, and tolerability of rimegepant in Japanese subjects for the prevention of migraine.
NCT05211154	Evaluation of the efficacy of diclofenac potassium and rimegepant for the acute treatment of migraine (atom)	Migraine, with or without aura	645	December 2025	The purpose of this study is to investigate whether 50 mg diclofenac potassium is non-inferior to 75 mg rimegepant in terms of pain freedom at 2 hours after drug intake.
NCT05156398	Efficacy and safety study of rimegepant for the preventative treatment of migraine in pediatric subjects	Migraine	640	September 2026	The purpose of this study is to compare the efficacy and safety of rimegepant to placebo as a preventative treatment for migraine in children and adolescents ≥ 6 to <18 years with episodic migraine.
NCT05198245	Study of pregnancy outcomes in women exposed to rimegepant during pregnancy	Migraine	4020	April 2028	The purpose of the study is to evaluate the risk of pregnancy and infant outcomes among women with migraine exposed to rimegepant during pregnancy and in two rimegepant unexposed comparator groups.
NCT05046613	Observational study to assess maternal, fetal and infant outcomes following exposure to rimegepant (monitor)	Migraine	780	April 2034	The purpose of the study is to evaluate fetal, maternal, and infant outcomes through 12 months of age.
UBROGEPANT					
NCT01657370	A pharmacokinetic study of mk-1602 in the treatment of acute migraine (mk-1602-007)	Migraine	195	December 2012	The purpose of this study is to characterize the pharmacokinetics of MK-1602 in the treatment of acute migraine, including the influence of demographic and other variables on MK-1602 pharmacokinetics, and to evaluate the relationship between MK-1602 concentrations and efficacy of the drug.

NCT01613248	A dose-finding study of mk-1602 in the treatment of acute migraine (mk-1602-006)	Migraine	834	December 2012	The purpose of this study is to assess the effectiveness, safety, and tolerability of a range of doses of MK-1602 versus placebo in the treatment of acute migraine.
NCT02828020	Efficacy, safety, and tolerability study of oral ubrogepant in the acute treatment of migraine (Achieve I)	Migraine, with or without aura	1672	December 2017	This study will evaluate the efficacy, safety, and tolerability of 2 doses of ubrogepant (50 and 100 mg) compared to a placebo for the acute treatment of a single migraine attack.
NCT02867709	Efficacy, safety, and tolerability of oral ubrogepant in the acute treatment of migraine (Achieve II)	Migraine, with or without aura	1686	February 2018	This study will evaluate the efficacy, safety, and tolerability of 2 doses of ubrogepant (25 and 50 mg) compared to a placebo for the acute treatment of a single migraine attack.
NCT02873221	An extension study to evaluate the long-term safety and tolerability of ubrogepant in the treatment of migraine	Migraine, with or without aura	1254	August 2018	This study will evaluate the long-term safety and tolerability of intermittent treatment with ubrogepant for the acute treatment of migraine over 1 year.
NCT04179474	Safety, tolerability and drug- drug interaction study of ubrogepant with erenumab or galcanezumab in participants with migraine	Migraine	40	December 2019	This study will evaluate the potential for a pharmacokinetic interaction and provide safety and tolerability information when ubrogepant and erenumab or ubrogepant and galcanezumab are co-administered.
NCT04818515	Study to assess adverse events and drug to drug interaction of oral tablet atogepant and ubrogepant in adult participants with a history of migraine	Migraine	26	June 2021	This study will assess the drug-to-drug interaction between atogepant and ubrogepant and assess the safety of atogepant and ubrogepant, when given alone or in combination, in adult participants with migraine.
NCT04492020	Study to evaluate oral ubrogepant in the acute treatment of migraine during the prodrome in	Migraine	1095	April 2022	Study to evaluate the efficacy, safety, and tolerability of oral ubrogepant in the acute treatment of migraine when administered during the prodrome.

	adult participants (ubr prodrome)				
NCT05264129	Study to assess adverse events when ubrogepant tablets in combination with atogepant tablets are used to treat adult participants with migraine	Episodic migraine	235	September 2023	This study will assess the safety and efficacy of the combined use of ubrogepant for the acute treatment of migraine headache in participants taking atogepant once daily for preventive treatment of migraine. Participants will receive oral atogepant tablets QD for 12 weeks followed by continued atogepant treatment with ubrogepant tablets taken as needed for the next 12 weeks.
NCT05214001	Evaluation of the efficacy of almotriptan and ubrogepant for the acute treatment of migraine (atom)	Migraine, with or without aura	645	December 2025	To investigate whether 12.5 mg almotriptan is non-inferior to 50 mg ubrogepant in terms of pain freedom at 2 hours after drug intake.
NCT05125302	Study to assess adverse events and disease activity of oral ubrogepant tablets for the acute treatment of migraine in children and adolescents (ages 6-17)	Migraine	1059	May 2026	The purpose of this study is to evaluate how safe and effective ubrogepant is in the acute treatment of migraine in children and adolescents. The study will include 2 cohorts of participants - PK cohort and main study (non-PK cohort). Participants aged 6-11 years in the PK Cohort will receive Dose A or Dose B of Ubrogepant for PK analysis to determine dose selection for the main study.
NCT05127954	Long-term extension study to assess safety and tolerability of oral ubrogepant tablets for the acute treatment of migraine in children and adolescents (ages 6-17)	Migraine	1200	March 2027	The purpose of this study is to evaluate the long-term safety and tolerability of ubrogepant in the acute treatment of migraine in children and adolescents.
NCT05158894	Observational study to assess adverse events when adult female participants are treated with ubrelvy (ubrogepant) during pregnancy	Migraine	1120	September 2034	The purpose of this study is to evaluate fetal, maternal, and infant outcomes through 12 months of age among women exposed to Ubrelvy during pregnancy, as well as in 2 Ubrelvy-unexposed comparison groups.
ATOGEPANT					

NCT02848326	Efficacy, safety, and tolerability of multiple dosing regimens of oral atogepant (agn-241689) in episodic migraine prevention	Migraine, with or without aura	834	April 2018	This study will evaluate the safety and tolerability of the following doses of atogepant (AGN-241689): 10 mg once daily (QD), 30 mg QD, 30 mg twice daily (BID), 60 mg QD, and 60 mg BID for the prevention of episodic migraine and will characterize the dose/response relationship.
NCT03700320	Study to evaluate the safety and tolerability of treatment with atogepant 60 mg daily for the prevention of migraine in participants with episodic migraine	Episodic migraine	744	May 2020	This study will evaluate the safety and tolerability of treatment with atogepant for the prevention of episodic migraine over one year.
NCT03777059	12-week placebo-controlled study of atogepant for the preventive treatment of migraine in participants with episodic migraine	Episodic migraine	910	June 2020	To evaluate the safety and tolerability of atogepant 30 mg and 60 mg once a day for the prevention of migraine in participants with episodic migraine.
NCT03939312	Extension study to evaluate the long-term safety and tolerability of oral atogepant for the prevention of migraine in participants with episodic migraine	Episodic migraine	685	March 2021	The purpose of this study is to evaluate the safety and tolerability of atogepant 60 mg once a day for the prevention of migraine in participants with episodic migraine.
NCT04818515	Study to assess adverse events and drug to drug interaction of oral tablet atogepant and ubrogepant in adult participants with a history of migraine	Migraine	26	June 2021	This study will assess the drug-to-drug interaction between atogepant and ubrogepant and assess the safety of atogepant and ubrogepant, when given alone or in combination, in adult participants with migraine. Participants will receive oral tablets of ubrogepant, followed by oral tablets of atogepant, followed by administration of oral tablets of atogepant and ubrogepant in combination. The study duration will be 30 days with a 7-day follow period.
NCT04829747	Study to assess adverse events (AEs) when oral	Chronic migraine	3	January 2022	The main objective of the study is to evaluate how safe and effective the atogepant is in preventing chronic migraine in adult Chinese participants who completed study 3101-303-

	atogepant tablet is given to adult chinese participants who completed study 3101-303-002 to prevent chronic migraine				002. Adverse events will be monitored. All participants will receive atogepant oral tablet once daily for 12 weeks.
NCT03855137	Efficacy, safety, and tolerability of atogepant for the prevention of chronic migraine	Chronic migraine	750	January 2022	This study will evaluate the efficacy, safety, and tolerability of atogepant in participants with chronic migraine. This study includes a 12-week treatment period.
NCT04740827	Atogepant for prophylaxis of migraine in participants who failed previous oral prophylactic treatments. (elevate)	Episodic migraine	300	August 2022	This study will assess the safety, tolerability, and efficacy of atogepant 60 mg compared with placebo in episodic migraines in participants who previously failed 2 to 4 classes of oral prophylactic treatments.
NCT05264129	Study to assess adverse events when ubrogepant tablets in combination with atogepant tablets are used to treat adult participants with migraine	Episodic migraine	235	September 2023	This study will assess the safety and efficacy of the combined use of ubrogepant for the acute treatment of migraine headache in participants taking atogepant once daily for preventive treatment of migraine. Participants will receive oral atogepant tablets once daily for 12 weeks followed by continued atogepant treatment with ubrogepant tablets taken as needed for the next 12 weeks.
NCT05216263	Study of oral atogepant when added to onabotulinumtoxina (botox) to assess adverse events and change in disease activity in adult participants with chronic migraine	Chronic migraine	125	October 2023	The study will assess the safety and tolerability of atogepant when added to BOTOX, as well as prospectively evaluate the efficacy of add-on atogepant for migraine prevention. All participants will receive atogepant oral tablet once daily during the 24-week treatment period, in addition to their standard of care Botox.
NCT04437433	A study evaluating oral atogepant for the prevention of migraine in japanese participants with	Chronic migraine; episodic migraine	170	February 2024	This study will evaluate the long-term safety, efficacy, and tolerability of atogepant 60 mg daily for the prevention of migraine in Japanese participants with chronic or episodic migraine.

	chronic or episodic migraine				
NCT04686136	A long-term safety and tolerability extension study evaluating atogepant for the prevention of chronic or episodic migraine	Episodic migraine; chronic migraine	670	August 2024	This study will evaluate the long-term safety and tolerability of atogepant 60 mg daily for the prevention of migraine in participants with chronic or episodic migraine
ZAVEGEPANT					
NCT04571060	Randomized trial in adult subjects with acute migraines	Migraine	1405	October 2021	The purpose of this study is to test the safety and efficacy of BHV-3500 versus placebo in the acute treatment of moderate or severe migraine.
NCT04408794	Long-term safety study of bhv-3500 (zavegepant*) for the acute treatment of migraine	Acute migraine	608	December 2021	The purpose of this study is to evaluate the long-term safety of BHV-3500/vazegepant intranasal in the acute treatment of migraine.
NCT04987944	Safety and efficacy active drug vs. placebo in subjects with asthma	Asthma	24	November 2022	This study is a double-blind, parallel-group, randomized study of active drug vs placebo in asthma.
NCT04346615	Safety and efficacy trial of zavegepant* intranasal for hospitalized patients with covid-19 requiring supplemental oxygen	COVID-19 infection	120	February 2023	The purpose of this study is to determine if a CGRP receptor antagonist may potentially blunt the severe inflammatory response at the alveolar level, delaying or reversing the path towards oxygen desaturation, ARDS, requirement for supplemental oxygenation, artificial ventilation, or death in patients with COVID-19 on supplemental oxygen.
NCT04804033	A study to evaluate the efficacy and safety of oral zavegepant in migraine prevention	Migraine	2900	July 2023	The purpose of this study is to compare the efficacy of BHV-3500 (zavegepant) to placebo as a preventive treatment for migraine, as measured by the reduction in the number of migraine days per month.

Supplementary Material 4 – Network meta‑analyses comparing the efficacy of lasmiditan, rimegepant, and ubrogepant published in the literature

Study		Agboola et al	Yang et al	Johnston et al	Polavieja et al
Year		2020	2021	2022	2022
Objective		Evaluate the health and economic outcomes of these three novel acute treatments for migraine attacks	To compare outcomes associated with the use of lasmiditan, rimegepant, and ubrogepant vs triptans for acute management of migraine headaches	Compare the relative efficacy and safety of rimegepant, ubrogepant, and lasmiditan for the acute treatment of migraine	Compare the relative efficacy and safety of rimegepant, ubrogepant, and lasmiditan for the acute treatment of migraine
Clinical trials included	Rimegepant	Study 301; Study 302; Study 303; Marcus et al (2014)	Study 301; Study 302; Study 303	Study 303	Study 301; Study 302; Study 303; Marcus et al (2014)
	Ubrogepant	ACHIEVE I; ACHIEVE II; Voss et al (2016)	ACHIEVE I; ACHIEVE II; Voss et al (2016)	ACHIEVE I; ACHIEVE II	ACHIEVE I; ACHIEVE II; Voss et al (2016)
	Lasmiditan	SAMURAI; SPARTAN; Färkkilä et al (2012)	SAMURAI; SPARTAN; Färkkilä et al (2012)	SAMURAI; SPARTAN	SAMURAI; SPARTAN; CENTURION; MONONOFU; Färkkilä et al (2012)
Considerations		This study included data about triptans (eletriptan, sumatriptan)	This study included data about triptans		
Main results	Efficacy/ potency (ascending order)	Rimegepant < ubrogepant < lasmiditan < triptans	Rimegepant = ubrogepant = lasmiditan > triptans	Ubrogepant < lasmiditan low-dose < rimegepant < lasmiditan high-dose	Ubrogepant, lasmiditan low-dose, rimegepant < lasmiditan high-dose
	Side effects	Lasmiditan had higher rates than other agents.	Lasmiditan had higher rates than other agents. Certain triptans (rizatriptan, sumatriptan, and zolmitriptan) were also associated with a higher risk of any adverse events when compared to rimegepant or ubrogepant	Lasmiditan had higher rates of somnolence and dizziness	Lasmiditan had higher rates of dizziness, fatigue, paraesthesia, sedation, nausea/ vomiting, and muscle weakness
	Discontinuation rate	Lower with ubrogepant and rimegepant	Higher discontinuation rate with triptans.	-	Analyses could not be performed for discontinuation due to adverse events (as a result of poor model fit)
Funding		Institute for Clinical and Economic Review	Ministry of Science and Technology, Taiwan	Biohaven Pharmaceuticals	Eli Lilly and Company

References:

1. Agboola F, Atlas SJ, Touchette DR, Borrelli EP, Rind DM, Pearson SD. The effectiveness and value of novel acute treatments for migraine. J Manag Care Spec Pharm 2020;26:1456-1462.
2. Johnston K, Popoff E, Deighton A, Dabirvaziri P, Harris L, Thiry A, Croop R, Coric V, L'Italien G, Moren J. Comparative efficacy and safety of rimegepant, ubrogepant, and lasmiditan for acute treatment of migraine: a network meta-analysis. Expert Rev Pharmacoecon Outcomes Res 2022;22:155-166.
3. Polavieja P, Belger M, Venkata SK, Wilhelm S, Johansson E. Relative efficacy of lasmiditan versus rimegepant and ubrogepant as acute treatments for migraine: network meta-analysis findings. J Headache Pain 2022;23:76.

4. Yang CP, Liang CS, Chang CM, Yang CC, Shih PH, Yau YC, Tang KT, Wang SJ. Comparison of New Pharmacologic Agents With Triptans for Treatment of Migraine: A Systematic Review and Meta-analysis. JAMA Netw Open 2021;4:e2128544.
5. Study 301: Lipton RB, Conway CM, Stock EG, Stock D, Morris BA, McCormack TJ, Frost M, Gentile K, Dubowchik GM, Coric V, Croop R. Efficacy, safety, and tolerability of rimegepant 75 mg, an oral CGRP receptor antagonist, for the treatment of migraine: results from a phase 3, double blind, randomized, placebo-controlled trial, Study 301. Presented at: the 60th Annual Scientific Meeting of the American Headache Society; June 28–July 1, 2018; San Francisco, CA, USA, Abstract 492562.
6. Study 302: Lipton RB, Croop R, Stock EG, Stock DA, Morris BA, Frost M, Dubowchik GM, Conway CM, Coric V, Goadsby PJ. Rimegepant, an Oral Calcitonin Gene-Related Peptide Receptor Antagonist, for Migraine. N Engl J Med 2019;381:142-149.
7. Study 303: Croop R, Goadsby PJ, Stock DA, Conway CM, Forshaw M, Stock EG, Coric V, Lipton RB. Efficacy, safety, and tolerability of rimegepant orally disintegrating tablet for the acute treatment of migraine: a randomised, phase 3, double-blind, placebo-controlled trial. Lancet 2019;394:737-745.
8. Marcus et al (2014): Marcus R, Goadsby PJ, Dodick D, Stock D, Manos G, Fischer TZ. BMS-927711 for the acute treatment of migraine: a double-blind, randomized, placebo controlled, dose-ranging trial. Cephalalgia 2014;34:114-25.
9. ACHIEVE I: Dodick DW, Lipton RB, Ailani J, Lu K, Finnegan M, Trugman JM, Szegedi A. Ubrogepant for the Treatment of Migraine. N Engl J Med 2019;381:2230-2241.
10. ACHIEVE II: Lipton RB, Dodick DW, Ailani J, Lu K, Finnegan M, Szegedi A, Trugman JM. Effect of Ubrogepant vs Placebo on Pain and the Most Bothersome Associated Symptom in the Acute Treatment of Migraine: The ACHIEVE II Randomized Clinical Trial. JAMA 2019;322:1887-1898.
11. Voss et al (2016): Voss T, Lipton RB, Dodick DW, Dupre N, Ge JY, Bachman R, Assaid C, Aurora SK, Michelson D. A phase IIb randomized, double-blind, placebo-controlled trial of ubrogepant for the acute treatment of migraine. Cephalalgia 2016;36:887-98.
12. SPARTAN: Kuca B, Silberstein SD, Wietecha L, Berg PH, Dozier G, Lipton RB; COL MIG-301 Study Group. Lasmiditan is an effective acute treatment for migraine: A phase 3 randomized study. Neurology 2018;91:e2222-e2232.
13. SAMURAI: Goadsby PJ, Wietecha LA, Dennehy EB, Kuca B, Case MG, Aurora SK, Gaul C. Phase 3 randomized, placebo-controlled, double-blind study of lasmiditan for acute treatment of migraine. Brain 2019;142:1894-1904.
14. CENTURION: Ashina M, Reuter U, Smith T, Krikke-Workel J, Klise SR, Bragg S, Doty EG, Dowsett SA, Lin Q, Krege JH. Randomized, controlled trial of lasmiditan over four migraine attacks: Findings from the CENTURION study. Cephalalgia 2021;41:294-304.
15. MONONOFU: Sakai F, Takeshima T, Homma G, Tanji Y, Katagiri H, Komori M. Phase 2 randomized placebo-controlled study of lasmiditan for the acute treatment of migraine in Japanese patients. Headache 2021;61:755-765.
16. Färkkilä et al (2012): Färkkilä M, Diener HC, Géraud G, Láinez M, Schoenen J, Harner N, Pilgrim A, Reuter U; COL MIG-202 study group. Efficacy and tolerability of lasmiditan, an oral 5-HT(1F) receptor agonist, for the acute treatment of migraine: a phase 2 randomised, placebo-controlled, parallel-group, dose-ranging study. Lancet Neurol 2012;11:405-13.
